# Pulmonary ossification: a case report

**DOI:** 10.3389/fmed.2025.1615806

**Published:** 2025-10-01

**Authors:** C. Di Somma, A. Annunziata, A. Coppola, G. E. Polistina, A. Marotta, G. Fiorentino

**Affiliations:** Unit of Pathophysiology and Respiratory Rehabilitation, Department of Intensive Care, A.O.R.N. Ospedali dei Colli, Naples, Italy

**Keywords:** hyperparathyroidism, calcification, ossification, pulmonary calcifications, CT scan

## Abstract

Pulmonary calcification and ossification are uncommon findings characterized by the deposition of calcium salts or bone tissue in the lung, generally associated with chronic kidney disease, hyperparathyroidism, and several other causes (1). Ossification is bone formation without or with marrow elements in the lung (1). In some patients, a new entity of subclinical primary hyperparathyroidism has been detected, characterized by persistently elevated parathyroid hormone concentration and normal serum calcium level, usually asymptomatic (2). CT scan is the standard method for detecting calcification and ossification. Radiological presentation on CT is characterized by round or lobulated nodules (3). Follow-up consists of monitoring lung images and lung function to assess disease progression. It suggests an annual CT scan. In this case, we presented a rare association of dendriform ossification with normocalcemic hyperparathyroidism, in particular, in a patient with pulmonary ossification, subclinical primary hyperparathyroidism, and diffuse calcifications.

## Introduction

Pulmonary calcification and ossification are uncommon findings characterized by the deposition of calcium salts or bone tissue in the lung, generally associated with chronic kidney disease, hyperparathyroidism, and several other causes (1). Ossification is bone formation without or with marrow elements in the lung (1). In some patients, a new entity of subclinical primary hyperparathyroidism has been detected, characterized by persistently elevated parathyroid hormone concentration and normal serum calcium level, usually asymptomatic (2). CT scan is the standard method for detecting calcification and ossification. Radiological presentation on CT is characterized by round or lobulated nodules (3). Follow-up consists of monitoring lung images and lung function to assess disease progression. It suggests an annual CT scan. In this case, we presented a rare association of dendriform ossification with normocalcemic hyperparathyroidism, in particular, in a patient with pulmonary ossification, subclinical primary hyperparathyroidism, and diffuse calcifications.

## Case presentation

A 77-year-old man who presented with a sustained dry cough and dyspnea for the past 8 months was admitted to the pneumology department. He smoked one pack of cigarettes a day for more than 25 years, until 32 years ago (25 pack-years). His medical history includes systemic arterial hypertension, tricuspid valve regurgitation, a healthy carrier of beta thalassemia, and polidistrectual osteoarthritis. Moreover, he reported diabetes mellitus type 2 since the age of 50, chronic kidney disease, nephrolithiasis, cholecystectomy surgery for gallstone disease 35 years ago, and salivary gland calcification. His therapy included olmesartan, metformin, and cardioaspirin.

Blood chemistry tests showed an elevated PTH (85.2 pg/mL, normal: 15–65 pg/mL), calcitonin serum level (11.8 pg/mL, normal: 0–5.5), normal corrected calcium (8.5 mg/dL), phosphorus serum level, high azotemia serum level (85 mg/dL, normal: 10–50 mg/dL) and vitamin D insufficiency. We detected the presence of serum and urinary free light chain (kappa: 72.65 mg/L; lambda: 34.66 mg/L; ratio K/L: 2.1; urinary chain K: 11.8 mg/L), normal urinary calcium excretion, and renal function was mildly impaired (eGFR 50 mL/min).

ABG and pulmonary functional tests were at standard limit values (FVC 91% predicted, FEV1 95% predicted, FEV1/FVC 79%; DLCO 60% predicted).

The patient underwent an ultrasound of the neck, which helped us to rule out primary hyperparathyroidism caused by adenoma or parathyroid gland hyperplasia.

Bronchoscopy with bronchoalveolar lavage revealed up to 83% neutrophils. Microscopy and culture tests were negative for *Mycobacterium tuberculosis,* and the QuantiFERON test was also negative. Esophagogastroduodenoscopy was performed, and gastroesophageal reflux disease was found.

**Figure 1:**
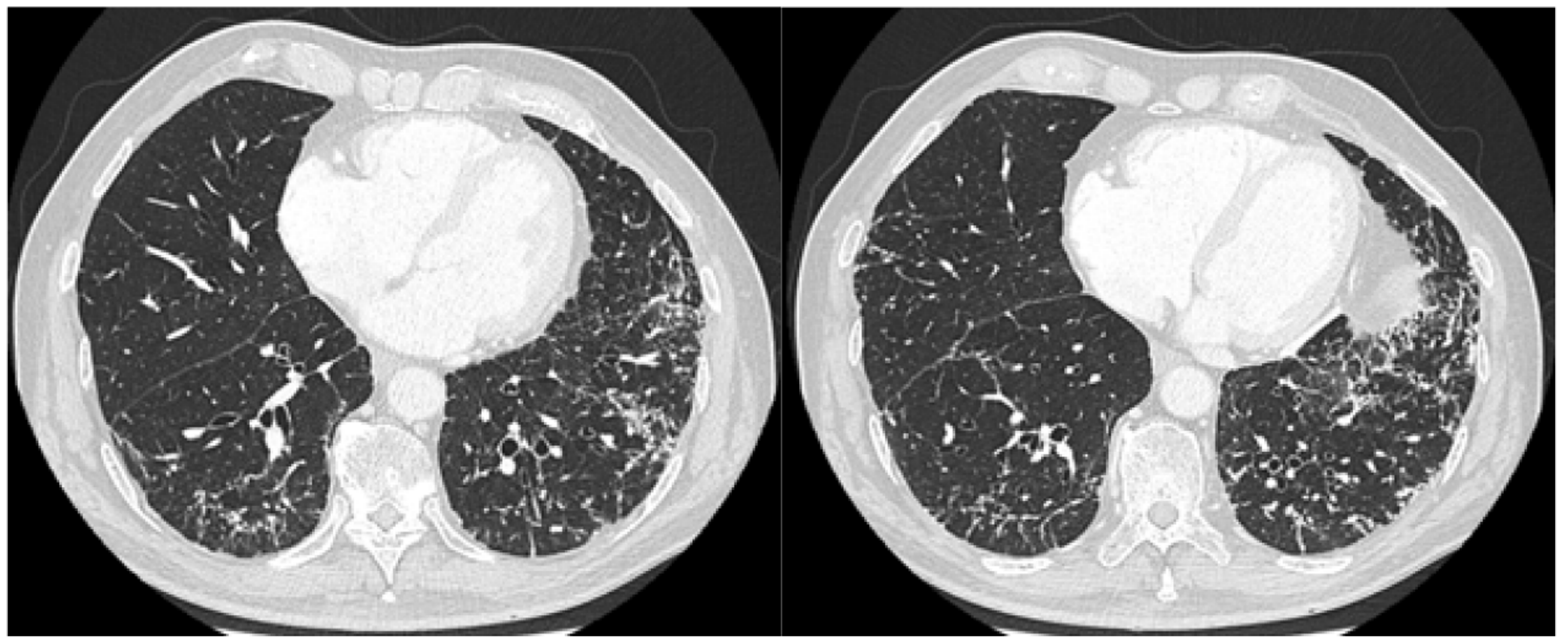
Lung CT scan: several micronodules, linear and branching, predominant in the lower lobes, with sublymphatic distribution and along the bronchovascular axis; most of these are calcific.

**Figure 2:**
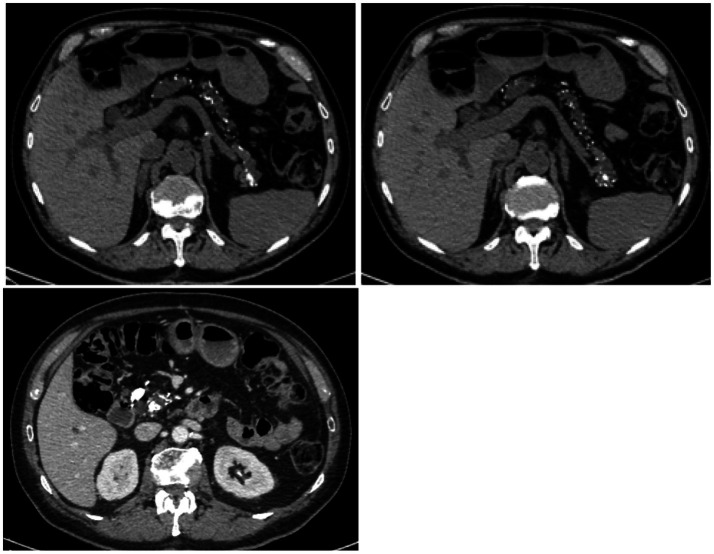
Abdominal CT scan: uneven and hypodense pancreas with disseminated calcifications, one of these in the pancreatic head.

High-resolution computed tomography of the chest showed a pattern of dendriform nodular ossification suggested by multiple micro-nodules, most calcific, with dominance in the lower lobes and micro-nodules organized in clusters and aggregates along interlobular septa resulting in a “coral like” form (Figure 1). Abdominal contrast CT scan showed irregular pancreas with diffuse calcifications (Figure 2). A 99mTc-MDP scan performed showed pathological accumulation of radiopharmaceutical.

Common diseases, such as pulmonary infection (tuberculosis, fungi, etc.), sarcoidosis, alveolar microlithiasis, and silicosis of pulmonary calcification, were excluded. Hematological diseases were excluded by specialists.

The patient was discharged with a diagnosis of the radiological pattern of dendriform lung ossifications, subclinical hyperparathyroidism, and multidistrict calcifications. He was treated with symptomatic therapy and vitamin D supplementation. The 2-year follow-up shows clinical and radiological stability.

## Discussion

Pulmonary calcification and ossification are common asymptomatic findings characterized by the deposition of calcium salts or bone tissue deposition in the lung. There are several causes: idiopathic, tumors, pneumoconiosis, infections, granulomatosis, heart disease, venous congestion conditions, and alterations in calcium and phosphorus metabolism (1).

Pulmonary calcifications, due to the precipitation of calcium salts in tissues with an alkaline environment, are divided into metastatic and dendriform. The metastatic form is more common in healthy tissues and is mostly linked to alterations in the metabolism of calcium and phosphorus. The most common risk factors are chronic renal failure, undergoing hemodialysis, and hyperparathyroidism. The deposition of calcium salts can occur in the alveolar spaces, interstitial septa, capillaries, and bronchial walls, with a predominance in the upper lung lobes. The alkaline environment is determined by an increased ventilation-perfusion ratio, which determines a reduced concentration of CO_2_ (4, 5). On imaging, they appear as centrilobular ground-glass opacities (GGO) and small nodules (3–10 mm) with peripheral calcifications. Pulmonary dendriform calcifications occur more frequently in previously damaged tissues, therefore secondary to infections or pneumoconiosis, and on imaging they appear as multiple large nodules >5 mm associated with signs of progressing parenchymal damage (3).

Pulmonary ossifications are characterized by the deposition of mature bone tissue within the alveolar or interstitial spaces, with tissue damage representing the main triggering factor. Currently, the pathogenesis is not completely clarified: the interaction of different factors, such as venous congestion, release of inflammatory cytokines, and growth factors, represents one of the possible causes of this entity. For these reasons, the greater flow of blood present in the lower lobes makes them more susceptible to damage. There is an idiopathic form, and they can be found in other lung diseases (idiopathic pulmonary fibrosis, amyloidosis, sarcoidosis, tuberculosis), cardiovascular diseases (mitral stenosis, heart failure, idiopathic hypertrophic subaortic stenosis), hypervitaminosis D, and primary and hyperparathyroidism. We can find nodular and dendriform ossification. The nodular forms are characterized by the deposition of bone tissue inside the alveolar spaces and appear as 1–4 mm circular calcific nodules, generally secondary to conditions of pulmonary venous congestion. In dendriform forms, ossification occurs at the pulmonary interstitium and septa and appears as fine, dendritic, branched calcifications with bronchovascular distribution, especially in the lower lobes, and is typical of idiopathic forms or secondary to states of chronic inflammation (1).

Most patients with pulmonary ossification and calcification are asymptomatic, and there is no correlation between the extent of calcifications and clinical symptoms. In secondary forms, the predominant symptoms are linked to the underlying pathology. Some might present restricted lung function, impaired diffusion capacity, and hypoxemia. Technetium 99 m labeled bone scanning radionuclides and HRCT of the thorax are complementary and allow a presumptive diagnosis of MPC, obviating the need for lung biopsy (6).

Treatment consists of treating the underlying causes in secondary forms and close follow-up for the idiopathic one. No clinical and radiological improvement has been observed after the use of corticosteroids or bisphosphonates.

Normocalcemic primary hyperparathyroidism (or subclinical primary hyperparathyroidism) is a newly defined entity characterized by an elevated parathyroid hormone concentration with a normal serum calcium level, after the exclusion of secondary hyperparathyroidism causes. This form is usually asymptomatic, but does seem to be a precursor of primary hyperparathyroidism. Clinical guidelines suggest that long-term surveillance might be appropriate in asymptomatic primary hyperparathyroidism (7).

The clinical history of our patient is represented by the presence of chronic renal insufficiency, which is supposed to have led to the onset of subclinical hyperparathyroidism. The kidney’s reduced production of the active metabolite of vitamin D leads to a reduction in calcium absorption at the intestinal level, with the stimulation of PTH production. At the same time, renal failure leads to a reduced excretion of calcitonin, with a consequent reduction in free calcium.

## Conclusion

From literature data, pulmonary calcifications and the development of diffuse calcifications are common in chronic primary hyperparathyroidism. In this case report, the presence of subclinical hyperparathyroidism could lead to the hypothesis of the presence of pulmonary calcifications, but the radiological characteristics of the chest confirm the presence of dendriform pulmonary ossifications. Furthermore, the concomitant tail of the pancreas calcifications could account for the presence of diabetes; on the other hand, the head one could explain gallstones.

The importance of this data is that, unlike pulmonary calcifications, several cases have been reported in the literature of the evolution of pulmonary ossifications into idiopathic pulmonary fibrosis (7, 8).

## Data Availability

The datasets presented in this study can be found in online repositories. The names of the repository/repositories and accession number(s) can be found in the article/supplementary material.
